# Visualization of the human enteric nervous system by probe confocal laser endomicroscopy: a first real-time observation of Hirschsprung’s disease and allied disorders

**DOI:** 10.1186/s12880-021-00648-7

**Published:** 2021-07-31

**Authors:** Atsushi Harada, Naoki Shimojima, Masakuni Kobayashi, Shunsuke Kamba, Seiichi Hirobe, Kazuki Sumiyama

**Affiliations:** 1 Department of Surgery, Tokyo Metropolitan Children’s Medical Center, 2-8-29 Musashidai, Fuchu, Tokyo, 183-8561 Japan; 2 Department of Endoscopy, The Jikei University School of Medicine, Tokyo, Japan

**Keywords:** Confocal laser endomicroscopy, Hirschsprung’s disease, Enteric nervous system, Ganglion cells, Transition zone

## Abstract

**Background:**

Our group previously proved that the human enteric nervous system can be visualized with confocal laser endomicroscopy after topical application of cresyl violet using surgically resected intestine specimens. The present report documents the first in vivo visualization of the human enteric nervous system with confocal laser endomicroscopy using local cresyl violet staining. The aim of this study was to evaluate the technical feasibility and clinical efficiency of confocal laser endomicroscopy in patients with Hirschsprung’s disease and allied disorders in vivo.

**Methods:**

Confocal laser endomicroscopy was performed in vivo in two patients to confirm the presence of the enteric nervous system during surgery in patients with Hirschsprung’s disease and allied disorders. Cresyl violet was gently injected from the serosal side into the muscular layer of the intestine, and scanning was performed within 30 min. Then, the scanned intestines were resected, and the visualized area of the specimens was pathologically evaluated.

**Results:**

The ganglion cell nuclei and the enteric nervous system network were clearly visualized intraoperatively in both cases. The morphological findings were similar to the pathological findings of the enteric nervous system in both cases although the period of visibility was brief.

**Conclusion:**

This study demonstrated the first, real-time observation of the enteric nervous system in humans using confocal laser endomicroscopy and suggest the potential to identify the enteric nervous system intra-operatively during surgery for Hirschsprung’s disease and allied disorders.

## Background

Confocal laser endomicroscopy (CLE) is a novel endoscopic technique that permits real-time, in vivo analysis of architectural and cellular details. Using this technique, cellular details below the tissue surface can be visualized at high magnification. In our previous ex vivo study, the enteric nervous system (ENS), especially the myenteric plexus (MP), was visualized in human intestinal specimens using CLE with fluorescent agents [1–4]. In the present study, cresyl violet (CV) was used as a fluorescent agent because of its high affinity to the ENS.

Hirschsprung’s disease is a congenital abnormality of the ENS in the distal intestine and requires resection of the abnormal colon segment (the aganglionic area) identified by intraoperative pathological analysis. The disorders allied to Hirschsprung’s disease are characterized by symptoms and signs similar to those of Hirschsprung’s disease, such as bowel obstruction, intestinal dilatation, and chronic constipation, despite the presence of ganglionic cells in the rectum [5]. The diagnosis of these diseases is based on a pathological evaluation of the frequency or morphology of ganglion cells in the affected intestinal segments. We therefore hypothesized that real-time visualization of the ENS by CLE might be a viable alternative to pathological diagnosis during surgery in patients with Hirschsprung’s disease and allied disorders.

In the present study, intraoperative CLE visualization for Hirschsprung’s disease and allied disorders was performed using a probe-based CLE system to confirm the technical feasibility of visualizing the ENS in patients with these diseases and to evaluate the clinical utility of the method.

## Methods

### The method of CLE visualization

**Fig. 1:**
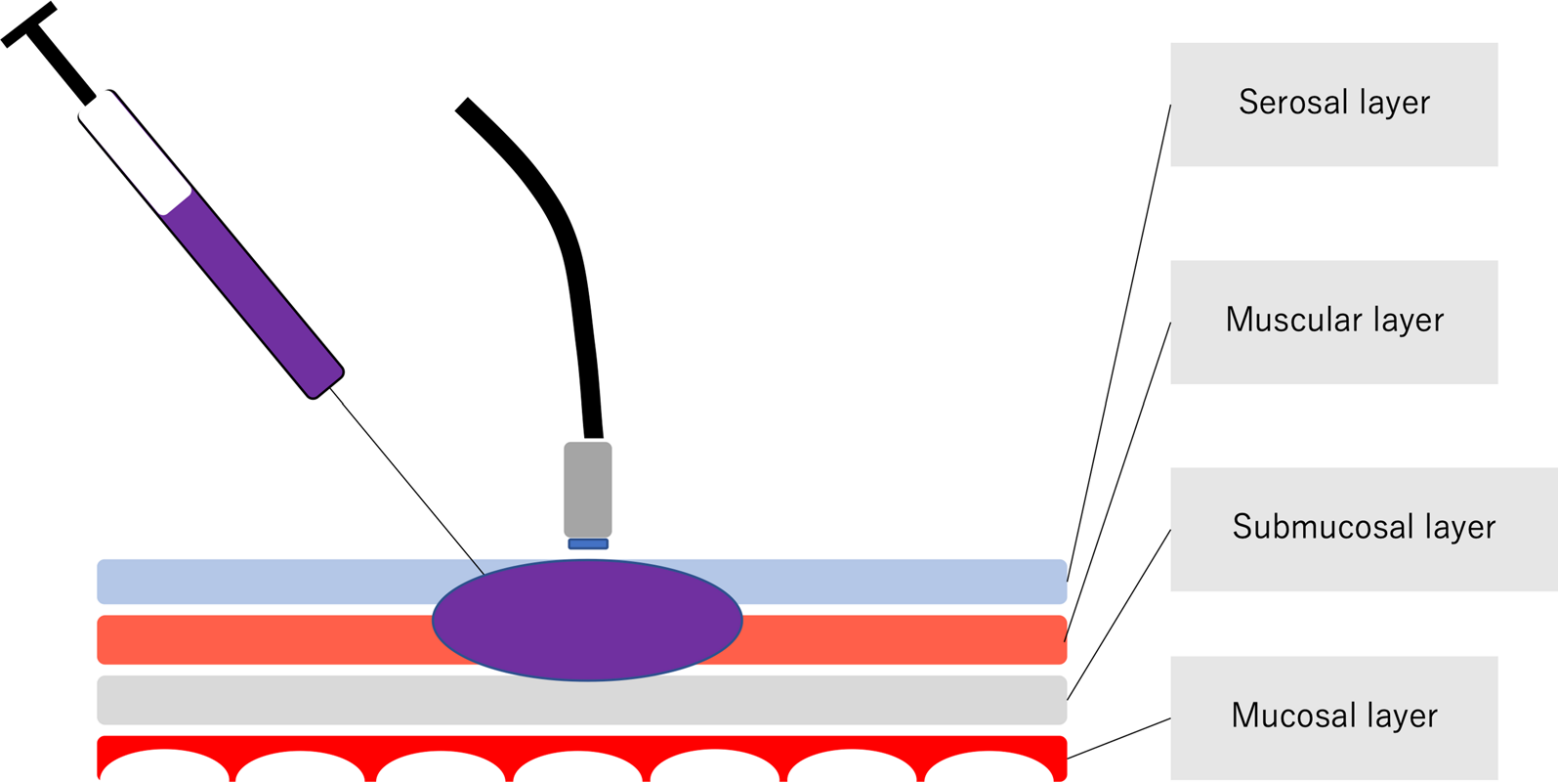
Crysel violet (CV) was injected locally until the serosal side of the intestines became grossly purple

CLE navigation was done to confirm the presence of the ENS by comparing the CLE findings with histological findings. Our observation of the target segments confirmed the presence of abundant ganglion cells although the procedure was not a surgical navigation using CLE to detect the segment to be resected but rather a trial evaluating the technical feasibility of the procedure during conventional surgery for Hirschsprung’s disease and allied disorders. CV (Muto Pure Chemical Co., Ltd., Tokyo Japan) was injected from the serosal side into the muscular layer for visualization by CLE. The dye was injected locally until the serosal side of the intestines became grossly purple (Fig. 1); then the intestines were immediately resected to minimize any possible adverse effect of the dye on the patients. The scanning time was kept to less than 30 min based on the average time required for a conventional intraoperative pathological diagnosis.

### Cresyl violet (CV)

CV is an organic compound with the chemical formula C_19_H_18_ClN_3_O. It is a basic dye commonly used in histology. CV was able to provide the clearest image of the ENS [1, 2] among several dyes tested, such as fluorescein, acriflavine [3], and NeuroTrace [6]. In addition, CV is apparently safe despite not being designed for use in the clinical setting. CV has been used widely in the histopathology lab for Nissl staining [7] or as a lysosomal marker [8]. Moreover, it has been used clinically for chromoendoscopies in Japan since the 1980’s and has an established safety profile [9–13]. CV has an emission and excitation wavelength of 630 and 585 nm, respectively [8], which the CLE device used in the present study can detect. Thus, CV was the best available dye for ENS visualization by CLE in the present study.

### The ethics and consent for clinical use of CV staining

As CV is not authorized for clinical use, careful attention was paid to designing the method of delivery into the intestinal muscle layers in vivo. First, CV was injected immediately before intestinal resection. Prior to its injection, the blood vessels in the mesentery were ligated and dissected to decrease the risk of CV circulation. Second, the observation time was kept to less than 30 min to decrease the risk of adverse effects. Third, the areas of the intestines into which CV was injected were resected in toto immediately after observation to minimize the amount of residual CV.

The aim and method of the present study, the potential risk of adverse effects, and the right to refuse participation prior to surgery were explained to the patients’ guardians, who gave their informed consent before enrollment. No complications were observed during or after the procedure in either case.

### Histological examination

All areas observed by CLE were pathologically evaluated, and the findings were compared against those of the CLE. All specimens were fixed with 10% formalin after surgical resection and sliced into horizontal sections because the surface of ENS visualization with CLE was shown horizontally. The thickness of each section was 4 μm. The ENS morphology observed in the pathological specimens stained with hematoxylin and eosin (HE) were then compared with the CLE findings. As CV was injected from the serosal side into the muscular layer until the intestines became grossly purple, we could easily detect almost the same part of visualization area even through specimens were fixed with formalin and sliced into horizontal sections.

### CLE system

GastroFlex-UHD (Mauna Kea Technologies, Paris, France) and CellVizio system (Mauna Kea Technologies, Paris, France) were used. The external diameter of the probe was 2.5 mm, the field-of-view of the obtained image was 240 μm in diameter, the imaging rate was 12 frames/s, and the image depth was 55-65 μm.

### Case 1

**Fig. 2:**
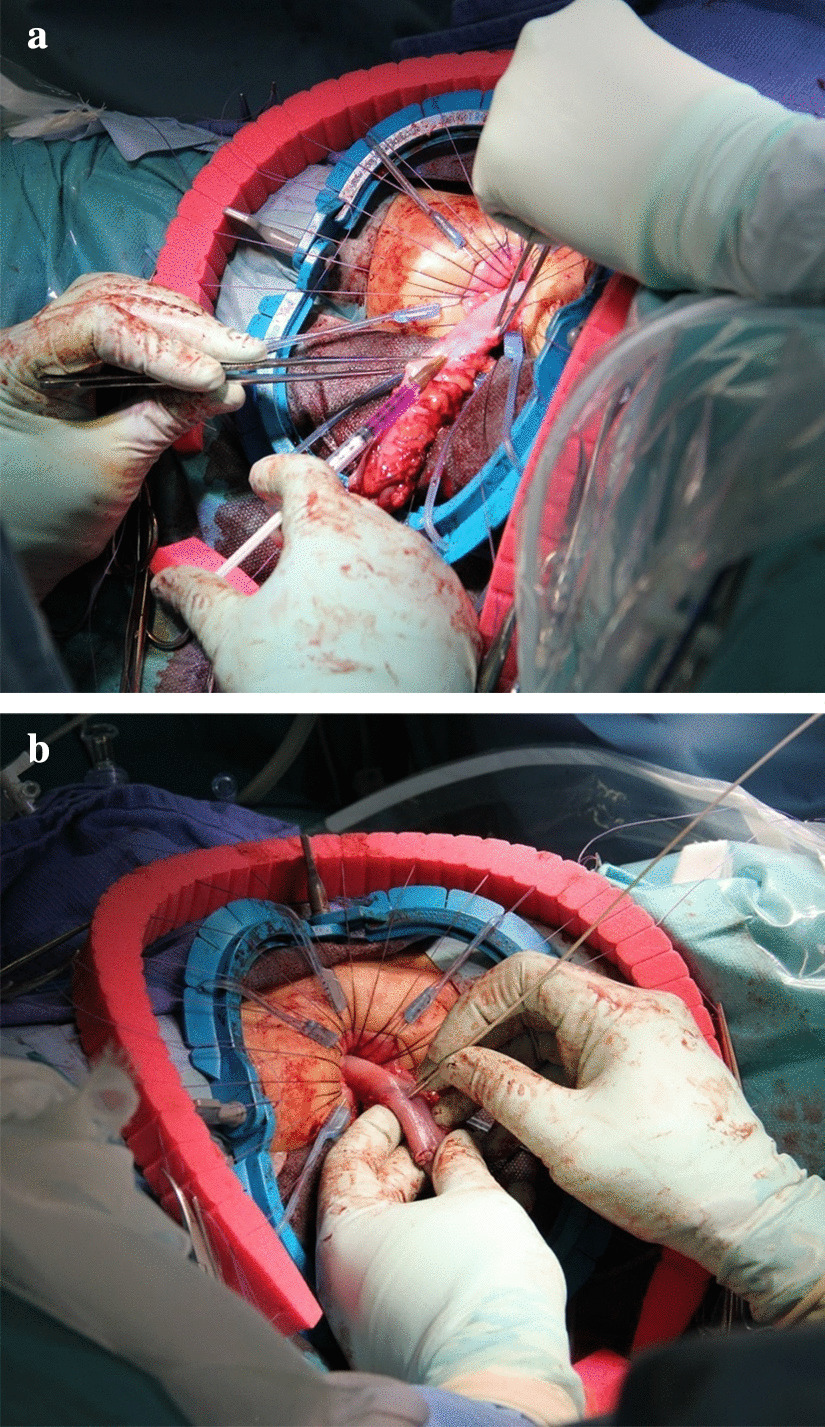
**a** Injection of CV during the pull-through procedure for Hirschsprung’s disease in Case 1. **b** Visualization of MP and ganglion cells by CLE from the serosal side

**Fig. 3:**
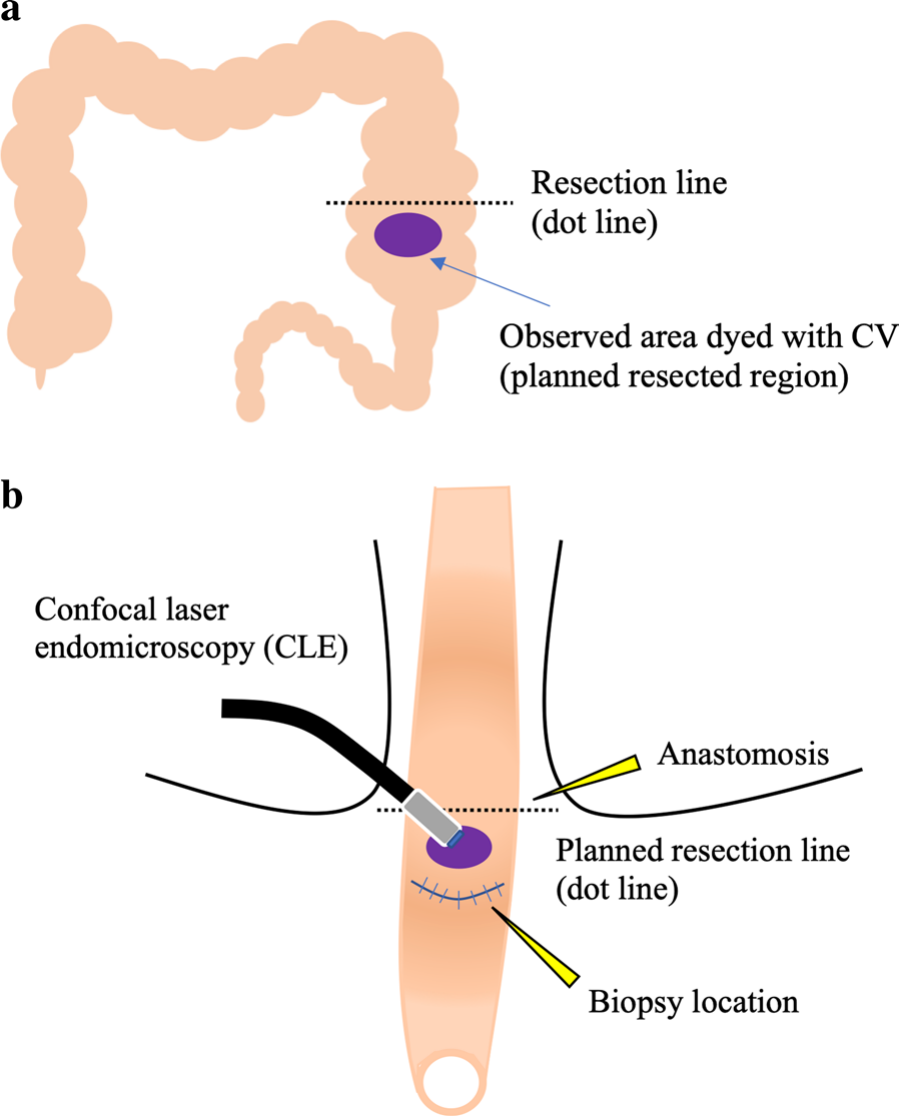
**a**, **b** CLE observation was performed just above the biopsy site of the colon in the resected region

The first case was that of a 2-year-old male patient with a past medical history of Lynn’s sphincteromyotomy for Hirschsprung’s disease at another hospital during the neonatal period. The chief complaint at his current presentation was constipation despite the previous operation. An enema revealed a stenotic region in the rectum. The pathological results of a rectal mucosal suction biopsy revealed the absence of ganglion cells with nerve fiber hypertrophy. The recto-anal inhibitory reflex (RAIR) on rectal manometry using a dilating balloon was also negative. Based on these findings, the short-segment type of Hirschsprung’s disease was diagnosed, and the endorectal pull-through procedure using the prolapsing technique was performed to resect the aganglionic segment. A laparotomy from the umbilical approach was performed, and an intraoperative pathological biopsy specimen of the intestine on the oral side 5 cm from the stenotic region in the rectum revealed abundant ganglion cells on later histopathological analysis of the frozen sections. Then, trans-serosal CLE observation was performed just above the biopsied region of the colon after a transanal mucosectomy immediately prior to the colon resection. CV was injected from serosal side (Fig. 2a), and CLE probe was attached gently to the colon dyed with CV to visualize the ENS (Fig. 2b). The scheme of observation area during surgery was shown in Fig. 3a, b.

### Case 2

**Fig. 4:**
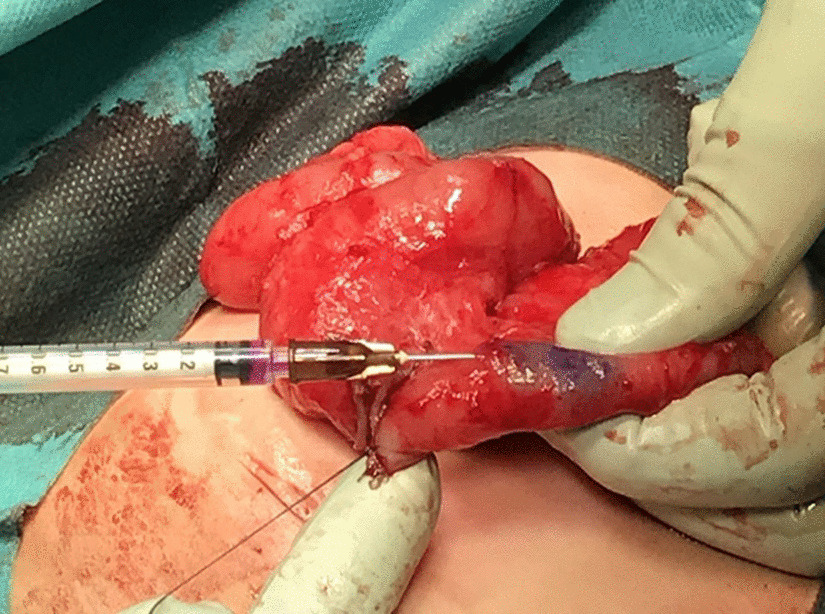
CV was injected from the serosal side into the muscular layer during stoma closure in Case 2

**Fig. 5:**
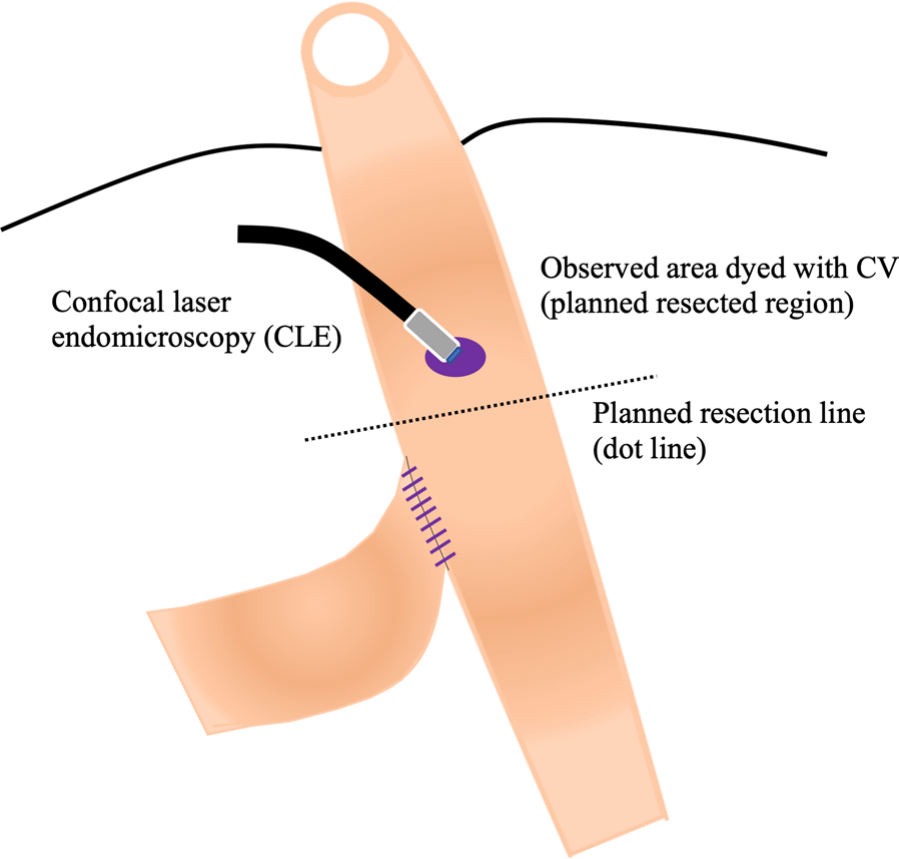
Visualization of ENS near the stoma on the anal side during Bishop–Koop enterostomy closure

The second case was that of a 1-year-5-month old female patient who received the diagnosis of allied disorder of Hirschsprung’s disease during the neonatal period. She was admitted to the hospital due to repeated, bilious vomiting on the day of birth. An ileostomy was placed on day 8, and a full-thickness biopsy of the ileum revealed an allied disorder of Hirschsprung’s disease. The pathology of the segment showed a markedly thickened muscular layer and solitary ganglion cells in the mucosa and intramuscular layer. Additionally, ganglion cells were observed in the MP. Based on these findings, an unclassified type of allied disorder of Hirschsprung’s disease was diagnosed. As intestinal motility improved with the patient’s development, a Bishop-Koop type enterostomy was placed. Defecation was achieved, and stoma closure was planned. Trans-serosal CLE observation was performed near the stoma on the anal side just before the resection and anastomosis (Fig. 4). Scheme was also shown in Fig. 5.

## Results

### ENS visualization by CLE and comparison with pathological findings

**Fig. 6:**
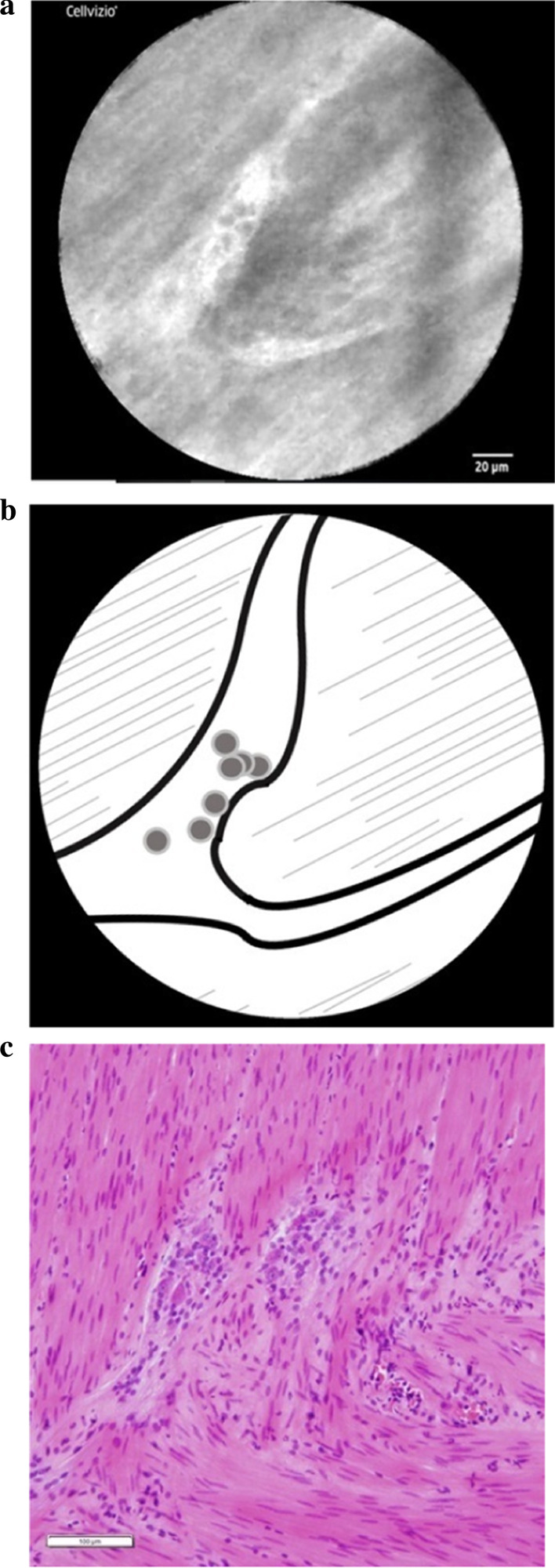
(**a**) The nuclei of the ganglion cells and the ENS network in the intestine were clearly visible in Case 1. (**b**) Scheme of these findings were shown. The ENS appeared as branches forming networks. The ganglion cells appeared as dark oval dots unstained by CV. (**c**) Morphologically similar ganglion cells were observed in the horizontal sections of intestine (HE staining. Magnification × 200)

**Fig. 7:**
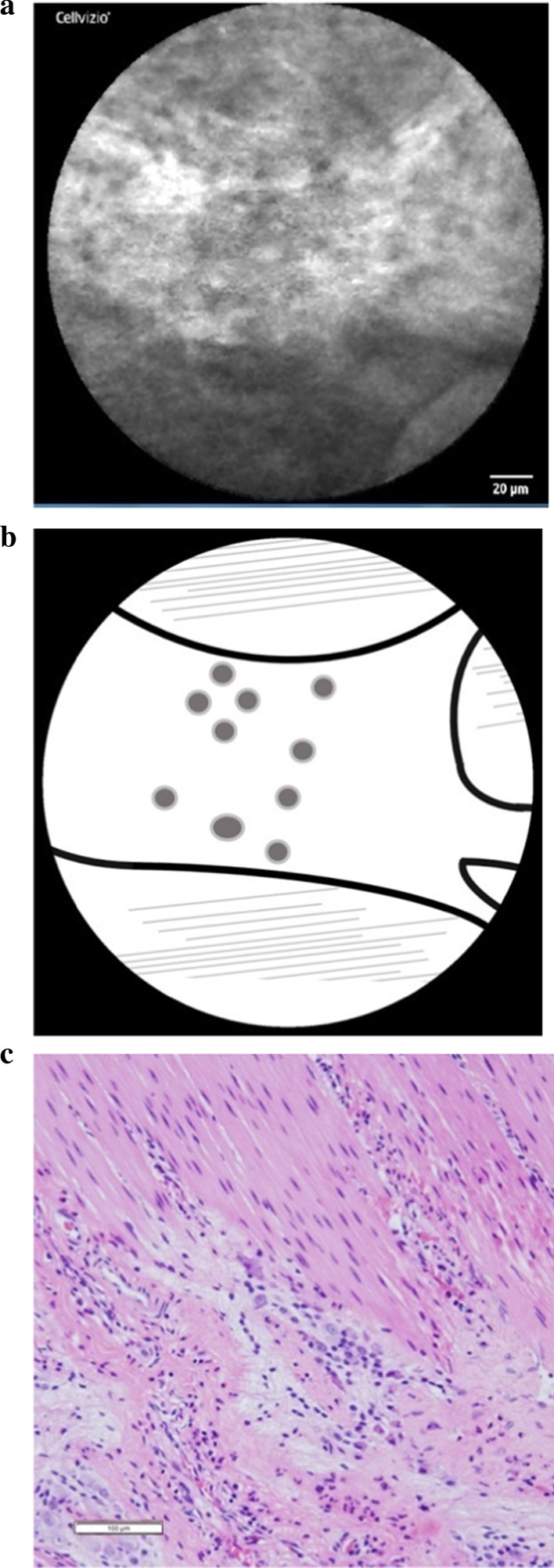
(**a**) The nuclei of the ganglion cells and the ENS network in the intestine were clearly visible in Case 2. (**b**) Scheme of these findings were shown. The ganglion cells appeared as dark oval dots unstained by CV. (**c**) Morphologically similar ganglion cells were observed in the horizontal sections of intestine (HE staining. Magnification × 200)

The ganglion cell nuclei and the ENS network were clearly observed by CLE during the operation in both patients, and a similarity in the morphology of the ganglion cells and ENS network was also noted in the pathological findings. (Case 1: Fig. 6a–c; Case 2: Fig. 7a–c). In Case 1, a forked neural network was able to be clearly observed, and several ganglion cell nuclei appeared as dark, oval dots in the plexus. In Case 2, a relatively thick and bright neural network appeared distinctly against the muscular layer in the background. The ganglion cell nuclei appeared as dark, oval dots in the plexus. The ENS network appeared as branches forming networks in both cases, and the ganglion cell nuclei appeared as dark, oval dots unstained by CV in line with the findings of a previous ex vivo study.

At first, CLE was unable to visualize the ENS from the serosa. The visualization of the ENS clearly improved after a slight incision was made to the serosa with an 18G needle in both cases although the total duration of visibility during scanning was brief. The maximum width of the nerve strands was 61.3 μm and 105.7 μm in the respective cases.

## Discussion

Real-time CLE visualization of the ENS is greatly advantageous for the treatment of Hirschsprung’s disease and allied disorders. The clinical utility of CLE consists in the real-time, intraoperative visualization of ganglionic cells and the ENS without the need for resection and in its application to a variety of GI functional disorders besides Hirschsprung’s disease and allied disorders, such as chronic constipation.

Hirschsprung’s disease is a congenital disorder of the ENS characterized by the absence of ganglion cells in the myenteric and submucosal plexuses of the distal intestine. The purpose of surgery for Hirschsprung’s disease is to excise the aganglionic bowel and reconstruct the intestinal tract. However, there are some problems with intra-operative pathological analysis. Although a preliminary biopsy is usually performed to measure the normal ganglionic bowel during surgery using a laparoscopic approach or a small umbilical incision, the procedure is time-consuming, and the specimen needs to be dyed and diagnosed by an expert pathologist during the operation as quickly as possible. In addition, pathologically evaluating the ENS accurately in a widespread region based on cross-sections of small specimens is difficult due to the transitional zone between the neuroanatomically normal and abnormal segments. Intraoperative, real-time CLE navigation may overcome these problems and enable accurate identification of the aganglionic segment to be resected without a preliminary biopsy during surgery.

The allied disorders of Hirschsprung’s disease are characterized by symptoms and signs similar to those of Hirschsprung’s disease, such as bowel obstruction, intestinal dilatation, and chronic constipation, despite the presence of ganglionic cells in the rectum [5]. There is no consensus on treatment as the disease is quite rare, and the etiology still remains unclear. Real-time CLE visualization of the ENS may have the potential to improve the treatment of this group of diseases.

The present study is the first trial of ENS visualization in Hirschsprung’s disease and allied disorders in vivo using CLE with CV staining. The study demonstrated the technical feasibility and clinical potential of confocal laser endomicroscopy in patients with these diseases to a certain extent. However, some challenges remain. Indeed, it was difficult to analyze the amount of ganglion cells or ENS quantitatively in the present device. When it comes to the diagnosis of the transition zone in Hirschsprung disease or Hirschsprung allied disorders, more quantitative analysis should be needed as this study was performed for only 2 cases and it is also the limitation of this study. Moreover, in both cases, the duration from CV injection to ENS staining was fairly long, making the procedure impracticable as a replacement for conventional intraoperative analysis at this juncture. Additionally, an incision in the serosa with an 18G needle was required in both cases because the image depth of the probe was too shallow at 55-65 μm to evaluate the muscular layer from the serosal side.

Samarasena [14] reported esophageal ENS visualization with needle-type CLE (nCLE: Mauna Kea Technologies, Paris, France) under endoscopic ultrasound (EUS) guidance. This technique was able to visualize the ENS without mucosal resection. An nCLE probe has a 325 mm field-of-view and an imaging depth of 40 to 70 μm. However, based on a previous ex vivo study, our group concluded that GastroFlex would be able to visualize the ENS more clearly than the nCLE in terms of depth, field-of-view, and resolution. In addition, the nCLE can only scan a limited range via a single needle puncture and requires several punctures. Therefore, we considered nCLE to be unsuitable for surgical navigation. The CLE system used in the study was designed for an esophagogastroduodenoscopy or colonoscopy. The CLE probe has a small diameter enabling it to be used with the conventional endoscopic accessory channel. During ENS observation, the probe was manually scanned by NS, who has experience in the procedure from a previous ex vivo study. Although the resected specimen was pinned on a corkboard for CLE scanning in the study, the scanned intestine in the present study was not fixed, rendering stable observation of the ENS more difficult. Our findings suggested that certain design modifications in confocal laser endoscopes, such as resizing for use during surgery, a wider field-of-view, and adjustable scanning depth, would be ideal for surgical navigation using CLE.

Furthermore, to achieve a successful neuro-navigation surgery for Hirschsprung disease, we have to deliver a fluorescence into ENS. In the current study, we injected CV as a fluorescent dye into the submucosal layer and detect it by CLE. We could successfully visualize the ENS structure but the area of scanning was limited. Our final goal is to visualize the ENS at a glance during surgery. And it should be safe for human use. In terms of the fluorescent dye, CV seemed to have good affinity to enteric neurons, but it was not neuron-specific and it had to be injected into each scan area. Neuron-specific marker with a certain fluorescent dye which can be delivered systemically or spraying irrigation in the abdominal cavity would be better.

In conclusion, the present study is the first to report ENS visualization using CLE in vivo and demonstrated the technical feasibility of visualizing the ENS in patients with Hirschsprung’s disease and allied disorders. Although much still remains to be done, we believe that success of ENS visualization in two live patients is a very important first step for the future, and this innovative, minimally-invasive, CLE navigation technique will contribute to improving conventional surgery for Hirschsprung’s disease and allied disorders.

## Data Availability

The data that support the findings of this study are available from the corresponding author, N.S, upon reasonable request.

## Abbreviations

CLE: Confocal laser endomicroscopy
ENS: Enteric nervous system
MP: Myenteric plexus
CV: Cresyl violet
HE: Hematoxylin and eosin
RAIR: Recto-anal inhibitory reflex
nCLE: Needle-type CLE
EUS: Endoscopic ultrasound
